# Supplementary material for: Minimal transmission in an influenza A (H3N2) human challenge-transmission model within a controlled exposure environment
Source: PLoS Pathog. 2020 Jul 13;16(7):e1008704. doi: 10.1371/journal.ppat.1008704 (PMC7390452; doi:10.1371/journal.ppat.1008704)
Supplement: S1 Text — (DOCX) [file ppat.1008704.s001.docx]

## S1 Appendix: EMIT Consortium Team Members

EMIT team members were: Walt Adamson, Blanca Beato-Arribas, Werner Bischoff, William Booth, Simon Cauchemez, Sheryl Ehrman, Joanne Enstone, Neil Ferguson, John Forni, Anthony Gilbert, Michael Grantham, Lisa Grohskopf, Andrew Hayward, Michael Hewitt, Ashley Kang, Ben Killingley, Robert Lambkin-Williams, Alex Mann, Donald Milton, Jonathan Nguyen-Van-Tam, Catherine Noakes, John Oxford, Massimo Palmarini, Jovan Pantelic, and Jennifer Wang. The Scientific Advisory Board members were: Allan Bennett, Ben Cowling, Arnold Monto, and Raymond Tellier.
