# Supplementary material for: Minimal transmission in an influenza A (H3N2) human challenge-transmission model within a controlled exposure environment
Source: PLoS Pathog. 2020 Jul 13;16(7):e1008704. doi: 10.1371/journal.ppat.1008704 (PMC7390452; doi:10.1371/journal.ppat.1008704)
Supplement: S2 Text — (DOCX) [file ppat.1008704.s002.docx]

**S2 Appendix: Role of Funding Source**

This research was funded as a cooperative agreement by the US CDC (<https://www.cdc.gov/publichealthgateway/partnerships/index.html>). Employees of the funder participated in discussion of the study design, and sample analysis; these individuals also participated in manuscript preparation and are named as co-authors. This work was also supported by the National Institute of Allergy and Infectious Diseases Centers of Excellence for Influenza Research and Surveillance (CEIRS) and this funder had no role in study design, data collection and analysis, decision to publish, or preparation of the manuscript.
