# Supplementary material for: Minimal transmission in an influenza A (H3N2) human challenge-transmission model within a controlled exposure environment
Source: PLoS Pathog. 2020 Jul 13;16(7):e1008704. doi: 10.1371/journal.ppat.1008704 (PMC7390452; doi:10.1371/journal.ppat.1008704)
Supplement: S3 Text — Contains Figs A-E. (DOCX) [file ppat.1008704.s003.docx]

# S3 Appendix: Efficacy of a Face Shield to Reduce Transmission of Influenza Virus in Large Droplets

(Werner Bischoff)

## Study Objective

The objective was to determine the efficacy of a face shield to selectively reduce transmission of large droplets containing viable influenza viruses without impeding droplet nuclei transfer in a mannequin model. The results of this study defined the feasibility of this intervention in selectively blocking such particles in a subsequent human-to-human influenza transmission study (EMIT).

## Rationale

Large particle (>10μm) behave as ballistic particles and will be stopped by a face shield while small particles (<5μm) can float over an extended period of time and distance and will be less effected by a face shield. It remains unknown how particles between 5 and 10μm will behave. Therefore, this study focused on the efficacy of a face shield in selectively blocking large particles from reaching the upper respiratory tract of human subjects while allowing small particles to enter.

## Methods

Established human aerosol dispersal patterns were used to evaluate the effect of a face shield in filtering out the large droplet fraction carrying influenza. For this purpose, we produced a range of particles sizes (<1 to >100μm) of a live Influenza virus (H1N1 Influenza virus A/WS/33) using an airbrush system. The carrier particle size was assessed in real time by an aerodynamic particle sizer (APS) for the particle range <1 to 20μm. Airborne virus was recovered by a six stage Andersen sampler with a styrofoam anatomical head placed on top allowing air to flow through a hose connecting the mouth opening to the sampler. The Styrofoam head allowed the anatomically correct positioning of the face shield to the mouth opening (Fisherbrand Full Face shield, Thermo Fisher Scientific Inc., Waltham, MA). Plaque forming units were counted by tissue culture plates (MDCK) for each sampler stage. The effect of a face shield was tested under the following conditions: absolute humidity (6.9 g/m3 [20°C, 40%RH]), air spray direction to face shield (straight, 90°), and air flow (none, 110ft/min). The face shield was modified during the trial to optimize small particle penetration while blocking large droplets. In addition to the virus exposure we also used latex beads of defined sizes (1, 5, 10, 15, 20, and 50μm) to conclusively determine the collection efficacy of our mannequin model. Beads were detected by flow cytometry (BD Accuri C6 system). Results are expressed in total counts and percent reduction of the viral/bead recovery load.

## Endpoint

The endpoint was the percent reduction in viral air load through a face shield by <20% for small particles <5μm (droplet nuclei) and >90% for large particles >10 μm (droplets). This was tested under the following conditions: air flow directions (straight vs. 90° turn, Fig A) and air velocities (no air movement vs. ~2.4 km/hour (Force 1 [Beaufort Scale description: light air] directed, turbulent airflow).

## Results

Wearing a face shield led to a 98.8% (straight; Fig B) and 97.6% (90° rotated; Fig C) reduction in large particles >4.7μm (p<0.05) compared with not wearing a face shield and facing straight toward the source. Small particles <4.7μm were not significantly affected (0.7% reduction [straight, Fig D], 10.6% reduction [90° rotated, Fig E]) (p>0.05) compared with not wearing a face shield and facing straight toward the source. Generating a directional air flow reduced large particles by 68.0% (straight, Fig B) and 99.4% (90° turn, Fig C) without face shield, and 98.7% (straight, Fig B) and 99.5% (90° turn, Fig C) with face shield (p<0.05). With directional airflow, small particles were reduced by 73.0% (straight, Fig D) and 54.6% (90° turn, Fig E) without face shield, and 83.9% (straight, Fig D) and 84.7% (90° turn, Fig E) with face shield.

**Fig A.** Straight and 90^o^ turn test conditions


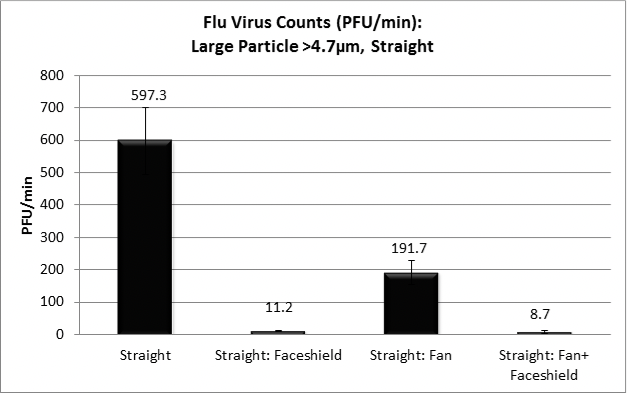


**Fig B*.*** Effect of Face Shield on Virus Transmission - Large Particle >4.7μm, error bars = standard deviation


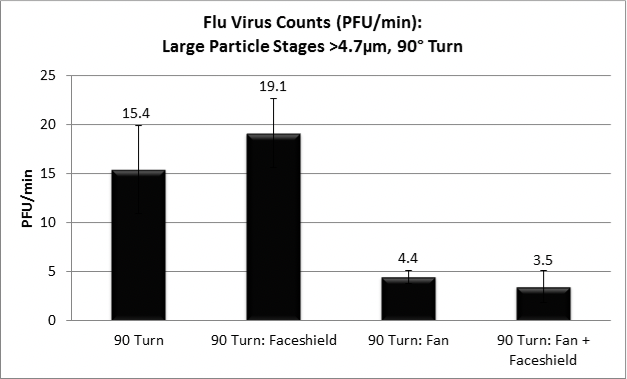


**Fig C.** Effect of Face Shield on Virus Transmission - Large Particles >4.7μm 90° Turn, error bars = standard deviation


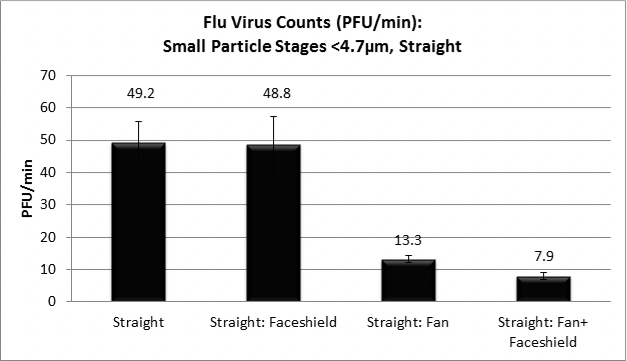


**Fig D.** Effect of Face Shield on Virus Transmission - Small Particles <4.7μm Straight Airflow, error bars = standard deviation


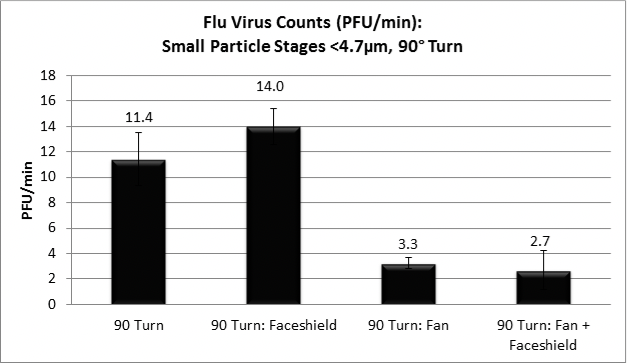


**Fig E.** Effect of Face Shield on Virus Transmission – Small Particles <4.7μm 90° Turn, error bars = standard deviation

The collection of beads was broken down by bead size and detection in the Andersen sampler stages for small particle <4.7μm and large particle stages >4.7μm. The face shield reduced 1 μm beads by 11.4% (straight) and 43.3% (90° turn) in the small particle stages and by 99.6% (straight) and 37.9% (90° turn) in the large particle stages. Directional airflow increased bead recovery by 185.4% in the small particle stages and reduced collection by 32.7% in the 90° head position. An overall reduction of 1μm beads was noted in the large particle stages (-98.3% [straight], -28.2% (90° turn). Only less than seven 5 μm beads per run were detected in the small particle stages as expected. In the large particle stages the face shield reduced 5μm bead collection by 7.9% (straight) and 77.1% (90° turn). Airflow led to a reduction by 12.5% (straight) and an increase of 17.8% (90° turn). Based on the expected absence of 5 μm bead findings in the smaller particle stages only the results of the large particle stages are reported for the 10, 15, 20, and 50μm bead sizes. In summary, the face shield successfully reduced beads by 95.2 to 100% (straight) and 23.3 to 87.7% (90° turn). Directional airflow led to a recovery reduction by 97.9-98.5% (straight) and 53.2-100% (90° turn).

## Conclusions

The face shield successfully blocked large particles >4.7μm from reaching the mouth and nose of a mannequin head while allowing passage of particles <4.7μm in a head-on air flow pattern in both virus and bead runs. Turning the head (90° rotation) significantly decreased the total virus detection compared to a head-on airflow pattern to very low concentrations (78.0-97.4%). Addition of a face shield while turned slightly increased the virus detection with face shield for both small and large particles (22-24%), while bead counts >4.7μm decreased (23-86%). The effects were not significant indicating negligible changes due to the face shield at the low recovery level. Increase of the air flow (turbulent) led to a decrease in virus detection in both small and large particles (straight and 90° rotated). With a head-on airflow 1 μm beads increased, however larger beads decreased (>90% for 10-50 μm beads). Combined with a 90° turn 1 to 50 μm beads decreased.

The modified face shield met the endpoint by reducing virus particles >10 μm by more than 90% while maintaining exposure to particles <5μm (<20% reduction). Turning the head perpendicular to the exposure source alone led to a substantial reduction of the overall virus recovery negating any significant effects of the face shield. The method of blocking selected particle sizes from reaching the human respiratory tract through a face shield is feasible and can be used to study virus transmission routes in human exposure studies.
