# Supplementary material for: Minimal transmission in an influenza A (H3N2) human challenge-transmission model within a controlled exposure environment
Source: PLoS Pathog. 2020 Jul 13;16(7):e1008704. doi: 10.1371/journal.ppat.1008704 (PMC7390452; doi:10.1371/journal.ppat.1008704)
Supplement: S4 Text — (DOCX) [file ppat.1008704.s004.docx]

# S4 Appendix: Full Inclusion and Exclusion Criteria, Power Calculation, and Sample Handling

## Inclusion criteria

- Age 18 to 45 years, inclusive.
- In good health with no history of major medical conditions from medical history, physical examination, and routine laboratory tests as determined by the Investigator by a screening evaluation.
- A total body weight ≥50 kg and a body mass index (BMI) >18 (if BMI is >32, a body fat percentage within WHO and NIH range for gender and age). BMI [kg/m^2^] = Body weight [kg] ÷ Height^2^ [m^2^].
- Non-sterilized males must agree to refrain from fathering a child from the point of entering Quarantine until the day 28 follow up visit by using an effective method of contraception.
- Sexually active females of child-bearing potential must agree to use 2 effective methods of avoiding pregnancy that are deemed to be effective from the point of entry into the Quarantine unit until the day 28 follow up visit.
- An informed consent document signed and dated by the subject and investigator.
- HAI titre ≤10 against challenge virus

## Exclusion criteria

- Subjects who have a significant history of any tobacco use at any time (≥total 10 pack
- year history, e.g. one pack a day for 10 years).
- Subjects who are pregnant or nursing, or who have a positive pregnancy test at any point in the study.
- Presence of any significant acute or chronic, uncontrolled medical illness (full list available on request), that in the view of the Investigator(s), is associated with increased risk of complications of respiratory viral illness.
- Abnormal pulmonary function in the opinion of the investigator as evidenced by clinically significant abnormalities in spirometry.
- History or evidence of autoimmune disease or known immunocompromise of any cause.
- Subjects with any history of asthma, COPD, pulmonary hypertension, reactive airway disease, or any chronic lung condition of any a etiology. The history of childhood asthma until and including the age of 12 is acceptable.
- Positive human immunodeficiency virus (HIV), hepatitis B (HBV), or hepatitis C (HCV) screen.
- Any significant abnormality altering the anatomy of the nose or nasopharynx.
- Any clinically significant history of epistaxis (nose bleeds).
- Any nasal or sinus surgery within 6 months of inoculation.
- Recent (within the last 3 years of the screening visit) and/or recurrent history of clinically significant autonomic dysfunction (e.g. recurrent episodes of fainting, palpitations, etc.).
- Any laboratory test or ECG which is abnormal and deemed by the investigator(s) to be clinically significant.
- Confirmed positive test for class A drugs or alcohol that cannot be satisfactorily explained (e.g. recent use of codeine tablets).
- Venous access deemed inadequate for the phlebotomy (and IV infusion) demands of the study.
- Subjects symptomatic with hayfever on admission into the unit for a quarantine session or prior to inoculation will be excluded.
- Any known allergies to the excipients in the challenge virus inoculums.
- Health care workers (including doctors, nurses, medical students and allied healthcare professionals) anticipated to have patient contact within two weeks of human viral challenge. Healthcare workers should not work with patients until 14 days after challenge or until their symptoms are fully resolved (whichever is the longer). In particular, any health care workers who work in units housing elderly, disabled or severely immunocompromised patients (e.g. bone marrow transplant units) will be excluded from participating in the study.
- Presence of household member or close contact (for an additional 2 weeks after discharge from the isolation facility) who:  is less than 3 years of age; has known immunodeficiency; is receiving immunosuppressant medication; is undergoing or soon to undergo cancer chemotherapy within 28 days of viral inoculation; has been diagnosed with emphysema or chronic obstructive pulmonary disease (COPD), is elderly and resides in a nursing home, or who has severe lung disease or another significant medical; has received a bone marrow or solid organ transplant
- Intending to travel within the next 3 months (to countries for which travel vaccinations are recommended).
- Those employed or immediate relatives of those employed at RVL or staff and students working directly in or for any of the units in which the Chief Investigator works.
- Receipt of blood or blood products, or loss (including blood donations) of 450 mL or more of blood, during the 3 months prior to inoculations.
- Acute use i.e. within 7 days prior to human viral challenge of any medication or other product (prescription or over-the-counter), for symptoms of hayfever, rhinitis, nasal congestion or respiratory tract infection.
- Receipt of any investigational drug within 3 months prior inoculation
- Receipt of more than 4 investigational drugs within the previous 12 months
- Prior participation in a clinical trial with the same strain of respiratory virus.
- Participation in any other respiratory virus challenge within 1 year prior to challenge.
- Receipt of systemic glucocorticoids, antiviral drugs, and immunoglobulins or any other cytotoxic or immunosuppressive drug within 6 months prior to dosing. Receipt of any systemic chemotherapy agent at any time.
- Presence of significant respiratory symptoms existing on the day of challenge or between admission for challenge and challenge with / exposure to virus.
- History suggestive of respiratory infection within 14 days prior to admission for challenge / exposure.
- Any other finding in the medical interview, physical exam, or screening investigations that, in the opinion of the investigator, GP or sponsor, deem the subject unsuitable for the study.

## Power Calculation

## Having achieved a secondary attack rate (SAR) of 25% in the ‘proof-of-concept’ study (1), we assumed that with increased numbers of donors per room, improved environmental control (temperature, humidity, ventilation rate), and a longer exposure time (increased from 2 days in the proof-of-concept study to 4 days) we would achieve an SAR of 40%. Based on a predicted SAR of 40% in CR and to detect a reduction of 50% (the magnitude of difference specified by the funder) in the IR group (i.e. a modified SAR of 20%), the statistical power of the overall experiment was estimated by conducting a computer simulation.

## Handling of Breath Samples

Concentration of fine aerosol samples and extraction of coarse aerosol samples were performed at the quarantine site. All samples were stored at -80˚C, shipped to the University of Maryland on dry ice, and stored at -80˚C until analysis.

**S4 Reference**

1. Killingley B, Enstone JE, Greatorex J, Gilbert AS, Lambkin-Williams R, Cauchemez S, et al. Use of a human influenza challenge model to assess person-to-person transmission: proof-of-concept study. J Infect Dis. 2012 Jan 1;205(1):35–43.
