# Supplementary material for: Minimal transmission in an influenza A (H3N2) human challenge-transmission model within a controlled exposure environment
Source: PLoS Pathog. 2020 Jul 13;16(7):e1008704. doi: 10.1371/journal.ppat.1008704 (PMC7390452; doi:10.1371/journal.ppat.1008704)
Supplement: S5 Text — Contains Table A. (DOCX) [file ppat.1008704.s005.docx]

**S5 Appendix: Baseline Characteristics**

Table A shows the baseline characteristics of the study volunteers, which were randomized as viral Donors (D), Intervention Recipients (IR), and Control Recipients (CR).

**Table A. Baseline Characteristics**

|  | D (n=52) | IR (n=40) | CR (n=35) |  |
| --- | --- | --- | --- | --- |
| **Gender** |  |  |  |  |
| Female | 15 (28.8%) | 13 (32.5%) | 12 (34.3%) |  |
| Male | 37 (71.2%) | 27 (67.5%) | 23 (65.7%) |  |
| **Age** |  |  |  |  |
| Median (Q1, Q3) | 30 (25, 38) | 28 (24, 34) | 27 (25, 36) |  |
