# Supplementary material for: Minimal transmission in an influenza A (H3N2) human challenge-transmission model within a controlled exposure environment
Source: PLoS Pathog. 2020 Jul 13;16(7):e1008704. doi: 10.1371/journal.ppat.1008704 (PMC7390452; doi:10.1371/journal.ppat.1008704)
Supplement: S6 Text — Contains Tables B-D. (DOCX) [file ppat.1008704.s006.docx]

# S6 Appendix: Comparing the Current Study and Proof-of-Concept Study Using Current Outcome Criteria and Applying Infection Criteria from The Proof of Concept Study

As shown in Results, the observed SAR in the current study was significantly below the expected 16% SAR that would have resulted from a doubling of the proof-of-concept SAR in response to design changes including doubling the number of Donors per Recipient and doubling the number of days of Recipient exposure to Donors. The observed SAR was also lower, but not significantly different from the 8.3% (1/12) observed in the proof of concept study, applying the main study infection criteria to the proof of concept study data (proof-of-concept v overall p = 0.26, proof-of-concept v CR p = 0.45, and proof-of-concept v IR p = 0.23).

The proof of concept study published by Killingley and colleagues (2012) used less stringent qRT-PCR-based infection classification criteria. Whereas the current study required two days of qRT-PCR positive nasopharyngeal swabs, the proof of concept study required just a single day of PCR or culture positive nasal wash or throat swab (1). Seroconversion criteria for infection was the same for both studies. None of the recipients from the proof of concept study were culture positive, two had a single day each of PCR positive nasal wash, and one had evidence of seroconversion. The recipients with single PCR positive washes did not seroconvert and the seroconversion did not have a positive wash. The PCR criteria were tightened because a single positive PCR, especially with high Ct values and without concomitant seroconversion, could represent random laboratory errors and because it is not clear that they represent true infection.

### Table B. qRT-PCR Results for Recipients with Sporadically Positive Tests.

|  |  | qRT-PCR C_t_ Value | |
| --- | --- | --- | --- |
| Volunteer ID | Study Day | Glasgow | CDC |
| 236 | 6 | No Amplification | 28.99 |
| 242 | 2 | No Amplification | 36.55 |

If we were to apply the proof-of-concept study’s infection classification criteria to the main quarantine transmission study, we would have observed 2 more infected donors and 2 more infected CR. Tables C and D reproduce Tables 1 and 3 from the manuscript main text (Donor and Recipient status, respectively), but apply the infection criteria used in the proof of concept study.

### Table C. Donor Status using infection criteria from proof of concept study

|  | | Clinical Illness (% of Infected) | | | Laboratory-confirmed Infection (% of Infected) | | |
| --- | --- | --- | --- | --- | --- | --- | --- |
| Quarantine # | Infected/Inoculated (%) | Symptomatic | Febrile | ILI | PCR-confirmed | PCR-confirmed & Seroconversion | Seroconversion by HAI : MN : Either |
| 1 | 15/20 (75) | 11 (73) | 4 (27) | 8 (53) | 13 (87) | 12 (80) | 12 : 14 : 14 |
| 2 | 11/12 (92) | 7 (64) | 0 (0) | 5 (45) | 11 (100) | 9 (82) | 9 : 7 : 9 |
| 3 | 18/20 (90) | 16 (89) | 2 (11) | 14 (78) | 17 (94) | 13 (72) | 14 : 11 : 14 |
| Total | 44/52 (85) | 34 (77) | 6 (14) | 27 (61) | 41 (93) | 34 (77) | 35 : 32 : 37 |

## Table D. Recipient Status using infection criteria from proof of concept study

|  |  |  | Clinical Illness  n (% of Exposed) | | | Laboratory-confirmed Infection  n (% of Exposed) | | |
| --- | --- | --- | --- | --- | --- | --- | --- | --- |
| Quarantine # | Recipient | Infected/Exposed n (%) | Symptomatic | Febrile | ILI | PCR-confirmed | PCR-confirmed & Seroconversion | Seroconversion by HAI : MN : Either |
| 1 | CR | 0/11 (0) | 4 (36) | 0 (0) | 3 (27) | 0 (0) | 0 (0) | 0 : 0 : 0 |
|  | IR | 0/10 (0) | 2 (20) | 0 (0) | 1 (10) | 0 (0) | 0 (0) | 0 : 0 : 0 |
| 2 | CR | 3/9 (33) | 2 (22) | 0 (0) | 2 (22) | 2 (22) | 0 (0) | 1 : 1 : 1 |
|  | IR | 0/10 (0) | 3 (30) | 0 (0) | 2 (20) | 0 (0) | 0 (0) | 0 : 0 : 0 |
| 3 | CR | 0/15 (0) | 6 (40) | 0 (0) | 4 (27) | 0 (0) | 0 (0) | 0 : 0 : 0 |
|  | IR | 0/20 (0) | 6 (30) | 0 (0) | 2 (10) | 0 (0) | 0 (0) | 0 : 0 : 0 |
| Total | CR | 3/35 (9) | 12 (34) | 0 (0) | 9 (26) | 2 (6) | 0 (0) | 1 : 1 : 1 |
|  | IR | 0/40 (0) | 11 (28) | 0 (0) | 5 (12) | 0 (0) | 0 (0) | 0 : 0 : 0 |

The original proof of concept reported an SAR of 25% (3/12). If we were to apply the less stringent, proof of concept infection criteria to the main study, we would observe an overall SAR of 4%, with three infections among CR and zero among IR. This represents a significant difference between the proof-of-concept and the main study overall (p = 0.03), in contrast to the non-significant difference between studies using the more stringent outcome criteria. The difference between the proof-of-concept and the CR group (p = 0.16 would continue to fall short of being statistically significant. However, the main study was designed to more than double the exposure and thus the expected SAR. Thus, in comparison with an expected SAR of 50%, the observed SAR was significantly lower (4% overall, p<0.0001; and 9% among CR, p<0.0001). The observed is similarly statistically significantly lower than the more conservative expected SAR of 40% used in the power calculations.

These alternate approaches to the outcome criteria produced similar results. Using the less stringent criteria increased the level of statistical significance showing that the difference between the larger challenge-transmission experiment and the proof-of-concept experiment was not driven by choice of outcome criteria.

**S6 Reference**

1. Killingley B, Enstone JE, Greatorex J, Gilbert AS, Lambkin-Williams R, Cauchemez S, et al. Use of a human influenza challenge model to assess person-to-person transmission: proof-of-concept study. J Infect Dis. 2012 Jan 1;205(1):35–43.
